# A Rice CPYC-Type Glutaredoxin *OsGRX20* in Protection against Bacterial Blight, Methyl Viologen and Salt Stresses

**DOI:** 10.3389/fpls.2018.00111

**Published:** 2018-02-09

**Authors:** Xi Ning, Yao Sun, Changchun Wang, Weilin Zhang, Meihao Sun, Haitao Hu, Jianzhong Liu, Ling Yang

**Affiliations:** Laboratory of Plant Molecular Physiology, College of Chemistry and Life Sciences, Zhejiang Normal University, Jinhua, China

**Keywords:** rice, glutaredoxin, *OsGRX20*, bacterial blight, methyl viologen, salt stress, genetic transformation

## Abstract

Glutaredoxins (GRXs) belong to the antioxidants involved in the cellular stress responses. In spite of the identification 48 *GRX* genes in rice genomes, the biological functions of most of them remain unknown. Especially, the biological roles of members of *GRX* family in disease resistance are still lacking. Our proteomic analysis found that OsGRX20 increased by 2.7-fold after infection by bacterial blight. In this study, we isolated and characterized the full-length nucleotide sequences of the rice *OsGRX20* gene, which encodes a GRX family protein with CPFC active site of CPYC-type class. OsGRX20 protein was localized in nucleus and cytosol, and its transcripts were expressed predominantly in leaves. Several stress- and hormone-related motifs putatively acting as regulatory elements were found in the *OsGRX20* promoter. Real-time quantitative PCR analysis indicated that *OsGRX20* was expressed at a significantly higher level in leaves of a resistant or tolerant rice genotype, Yongjing 50A, than in a sensitive genotype, Xiushui 11, exposed to bacterial blight, methyl viologen, heat, and cold. Its expression could be induced by salt, PEG-6000, 2,4-D, salicylic acid, jasmonic acid, and abscisic acid treatments in Yongjing 50A. Overexpression of *OsGRX20* in rice Xiushui 11 significantly enhanced its resistance to bacterial blight attack, and tolerance to methyl viologen and salt stresses. In contrast, interference of *OsGRX20* in Yongjing 50A led to increased susceptibility to bacterial blight, methyl viologen and salt stresses. *OsGRX20* restrained accumulation of superoxide radicals in aerial tissue during methyl viologen treatment. Consistently, alterations in *OsGRX20* expression affect the ascorbate/dehydroascorbate ratio and the abundance of transcripts encoding four reactive oxygen species scavenging enzymes after methyl viologen-induced stress. Our results demonstrate that OsGRX20 functioned as a positive regulator in rice tolerance to multiple stresses, which may be of significant use in the genetic improvement of rice resistance.

## Introduction

A variety of environmental stresses, including pathogen attack, photooxidation, salinity, abnormal temperature, and drought, stimulate the production of reactive oxygen species (ROS). Excessive ROS can induce macromolecular damage and change normal signal conduction (Fukao et al., 2011; Laporte et al., 2012). To cope with such oxidative toxicity, plant cells have developed precisely controlled anti-oxidative systems and maintain a reduced intracellular state with the help of redox-controlling proteins (Lee et al., 2002). Glutaredoxins (GRXs) belong to the ubiquitous oxidoreductase enzymes (Rouhier, 2010; Sharma et al., 2013), and have the ability to reduce dehydroascorbate (DHA), peroxiredoxin, and methionine sulfoxide reductase, thereby contributing to the removal of ROS and the repair of oxidative damage of macromolecules (Sha et al., 1997; Morita et al., 2015). A high number of *GRX* genes were identified in plant genomes. Plant GRXs have been divided into three classes based on amino acid sequences of the active-site motif: CGFS-, CPYC- and CC-type (Rouhier, 2010). In spite of the identification of 48 *GRX* members in rice based solely on genome analyses, the biological functions and physiological roles of most of them remain unknown (Garg et al., 2010). It has been reported recently that overexpression of a CC-type *GRX* gene, *OsGRX8*, in *Arabidopsis* plants reduced sensitivity to auxin and stress hormone, abscisic acid (ABA), and increased tolerance to various abiotic stresses, including oxidative stress, osmotic and salinity. The knock-down of *OsGRX8* made the RNA-interfering (RNAi) seedlings more susceptible to the aforementioned abiotic stresses (Sharma et al., 2013). *MICROSPORELESS1* (*MIL1*) encoding a meiocyte centromere-localized CC-type GRX acted as interacting partners of TGA transcription factors and regulated meiosis initiation in microsporocytes within rice anther (Hong et al., 2012). Concerning the CPYC-type GRXs in rice, only one of the seven members has been characterized, namely *OsGrxC2;2* (Sha et al., 1997; Morita et al., 2015) or *OsGRX14* (Garg et al., 2010). OsGrxC2;2 was localized in subcellular compartments of the secretory pathway including the plasma membrane, Golgi complex, and endoplasmic reticulum (Morita et al., 2015). OsGrxC2;2 with a CPFC active site possess the activities of dehydroascorbate reductase (DHAR) and thioltransferase (Sha et al., 1997), and also demonstrates glutathione (GSH)-dependent peroxidase activity (Lee et al., 2002). The *OsGrxC2;2* transcript was induced by methyl viologen (MeV) treatment together with the superoxide dismutase (SOD) and type-*h* thioredoxin (TRX) genes, suggesting that OsGrxC2;2 is involved in oxidative stress defense (Tsukamoto et al., 2005). OsGrxC2;2 protein in developing seeds accumulates particularly in the aleurone layers and participates in the tolerance to oxidative stress in developing and mature seeds (Morita et al., 2015). However, the roles of members of *GRX* family in disease resistance remain unknown.

Bacterial blight, caused by *Xanthomonas oryzae* pv. *oryzae* (*Xoo*) is one of the most serious bacterial disease of rice worldwide. Our proteomic analysis demonstrated that a putative auxin-regulated protein (GenBank Accession No. XP_483757; RAP-DB gene ID Os08g0558200) increased by 2.7-fold after infection by *Xoo* strain-Zhe173 (Yu et al., 2008). We further cloned the full-length gene and found that it encoded a protein for CPYC-type GRX, OsGRX20 (Garg et al., 2010). A member of rice CPYC-type GRX class, *OsGrxC2;2*, was shown to function in the tolerance to oxidative stress in developing and mature seeds (Morita et al., 2015). Therefore, we postulated that the *OsGRX20* expressed in rice leaves plays a role in resistance to *Xoo* and tolerance to multiple oxidative stresses, and characterize its biological functions by transgenic approaches.

## Materials and Methods

### Plant Materials and Growth Condition

Beneficial genes of the wild rice *Oryza meyeriana* Baill were introduced into a *japonica* rice cultivar Xiushui 11, former name used as 8411, through asymmetric somatic hybridization (Yu et al., 2008). A stable hybrid line Yongjing 50A, former name SH5 (BC_1_F_12_, backcrossed with Xiushui 11), exhibits the broad-spectrum resistance to *Xoo* strains, while its backcross parent Xiushui 11 is less resistant (Yu et al., 2008; Zhang et al., 2015). Rice seedlings were grown in nutrient solution under a 16/8 h light/dark cycle at a light intensity of 350–400 μmol m^-2^ s^-1^ at 28/25°C day/night. The seedling roots at five-leaf stage were exposed to heat (45°C), cold (4°C), high salinity (150 mM NaCl), 20% PEG-6000, and MeV (10 μM or 20 μM). The seedling leaves per tank were sprayed with 100 mL of 100 μM MeV, 20 μM 2,4-D, 2 mM salicylic acid (SA), 100 μM jasmonic acid (JA), and 100 μM ABA in 0.02% Tween-20, while mock controls were sprayed with 0.02% Tween-20 solution without addition of MeV or growth regulators. The fully expanded uppermost leaves were inoculated with *Xoo* strain Chinese pathotype IV (Zhe173) by the leaf-clipping method (Yu et al., 2008). *Nicotiana benthamiana* plants were grown in a growth room under a 16-h light/8-h dark cycle at 25/23°C day/night.

### Isolation and Analysis of Full-Length *OsGRX20*

Total RNA was extracted from 2 g of leaf, root or young stem tissues with TRIzol reagent. First-strand cDNA was synthesized from 1 μg of total RNA using the Superscript II Kit (Invitrogen, Carlsbad, CA, United States). A pair of primers GRX20-F and GRX20-R (Supplementary Table S1) was designed based on the sequence of GenBank accession No. XP_483757. The full-length genomic DNA was amplified by using the Fast HiFidelity PCR Kit using the same primers as described above and genomic DNA from leaves.

### Gene Expression Analysis by Quantitative Real-Time PCR (qPCR)

The primers GRX20q-F and GRX20q-F (Supplementary Table S1) were used to amplify 268 bp of *OsGRX20*. The qPCR analysis was performed as described (Zhang et al., 2015). PCR was run in a StepOnePlus (Applied Biosystems, Foster City, CA, United States). The gene *β*-actin (X15865) was used as reference. Two independent biological replicates and three technical replicates were used for the analyses.

### Vector Construction and Rice Transformation

To construct the *OsGRX20* promoter *β*-glucouronidase (GUS) reporter vector, a 2501-bp DNA fragment upstream of the start codon was amplified by using a pair of primers GRX20p-F and GRX20p-R (Supplementary Table S1) from Yongjing 50A genomic DNA and then inserted into the 5′ end of *GUS* gene of the binary expression vector pCAMBIA1391Z.

To overexpress the *OsGRX20* gene, a 1026-bp cDNA fragment was amplified with specific primers GRX20OE-F and GRX20OE-R (Supplementary Table S1). The PCR product was double digested by *Kpn*I and *Xba*I and ligated into the binary vector pCAMBIA1301 driven by the CaMV35S promoter.

For the RNAi construct, two 341-bp fragments corresponding to the specific sequence of *OsGRX20* ORF were amplified using primers GRX20SE-1F and GRX20SE-1R for 5′ sense orientation, GRX20SE-2F and GRX20SE-2R (Supplementary Table S1) for 3′ antisense orientation. The PCR products were ligated into both sides of the second intron of *NIR1* gene in pBS-in vector. The fragment was inserted into the pCAMBIA1301 vector by digestions.

The recombinant plasmid was mobilized into *Agrobacterium tumefaciens* strain EHA105 by heat shock method. The overexpression vector was transformed into rice var. Xiushui 11 using calli induced from mature embryos by *Agrobacterium*-mediated method, and the RNAi vector was used to transform into Yongjing 50A. Transformed calli were selected for 25 mg mL^-1^ hygromycin resistance for 4 days. PCR screening of transgenic seedlings was performed using specific primers of the *OsGRX20* cDNA to select homozygous lines.

### Histochemical Analysis and GUS Assay

Transgenic rice samples were incubated in GUS staining buffer (100 mM NaH_2_PO_4_ pH 7.0 containing 0.5% Triton X-100, 20% methanol, 1 mM K_3_[Fe(CN)_6_], 1 mM K_4_[Fe(CN)_6_] and 0.5 mg mL^-1^ X-Gluc) overnight at 37°C. Organs were rinsed and fixed in formalin acetic acid ethanol solution at 4°C overnight, subsequently observed and photographed using an Olympus SZX16 (Tokyo, Japan).

### Generation of Fusion Proteins and Subcellular Localization

The entire coding region of OsGRX20 was amplified by using primers GRX20OE-F and GRX20s-R (Supplementary Table S1). Validated cDNA inserts in the pMD-18T vector were cloned into the pCAMBIA1300-35S-eGFP (no ATG) fusion vector at the 5′-end of the *GFP* gene by single digestion with *Kpn*I. The resulting fusion construct (35S:GFP:*OsGRX20*) and the control vector (35S-GFP) were introduced into *A. tumefaciens* strain GV3101, while empty vector 35S:RFP into *A. tumefaciens* strain GV2260. Transiently expression in *N. benthamiana* leaves via agroinfiltration as described by Yang et al. (2016). Images were captured by a confocal laser scanning microscopy (Leica TCS SP5 AOBS, Wetzla, Germany). Excitation wavelengths were 488 nm for GFP and 550 nm for RFP. Emission was detected at 505–530 nm for GFP and 570–610 nm for RFP.

### *Xoo* Inoculation and Disease Evaluation

The most destructive *Xoo* strain, i.e., Philippine race 6 (PXO99) was used to evaluate the resistance to *Xoo* disease. Bacterial inoculum was prepared from 48 h culture on potato sucrose agar slants and its density was adjusted to 10^9^ colony-forming units mL^-1^. The fully expanded flag leaf at the booting stage was inoculated by the leaf-clipping method (Yu et al., 2008). The eight plants in the middle of each row were used for measuring and disease resistance was evaluated by the percentage of lesion length/leaf length (i.e., lesion area) at the 21st day post inoculation (Ding et al., 2008).

### Chlorophyll Content

Chlorophyll relative content was measured on the 7th day after treatment by a SPAD-502 Chlorophyll Meter (Konica Minolta, Beijing, China). Each leaf was measured at least three times, and the relative chlorophyll content was calculated based on the values of 18 leaves.

### Determination of Superoxide Radical, Ascorbate (ASC) and DHA Levels

To detect the level of superoxide radicals in seedlings of wild-type and transgenic lines, the hydroxylamine oxidation-based assay was used (Bors et al., 1977). The levels of ASC and DHA in leaves were quantified as described (Gillespie and Ainsworth, 2007).

### Statistical Analysis

Each value represents the mean ± SD from at least three biological replicas. Student’s *t*-test was used to measure statistical significance. Significant results are marked with asterisk (^∗^*p* < 0.05 and ^∗∗^*p <* 0.01).

## Results

### Isolation and Characterization of *OsGRX20*

Our previous studies have demonstrated that rice variety Yongjing 50A (former name SH5) exhibits the broad-spectrum resistance to *Xoo* strains, while Xiushui 11 (former name 8411) is susceptible to *Xoo* strains (Huang et al., 2008; Zhang et al., 2015). The cloned cDNA sequence of *OsGRX20* gene from Yongjing 50A was identical with that from Xiushui 11, which was 1302 bp in length and contained a 1026-bp ORF encoding 341 amino acids. The calculated molecular mass of the protein was 37.22 kDa, which was confirmed by SDS-polyacrylamide gel analysis of heterologous expression of *OsGRX20* in *Escherichia coli*. Subsequently, the genomic region of the *OsGRX20* was amplified from both Xiushui 11 and Yongjing 50A, and DNA sequences of two 2568-bp fragments were exactly the same. Sequence analysis revealed that the 2580-bp full-length *OsGRX20* comprised 12 exons and 11 introns, which differed from the predicted results (Garg et al., 2010).

Multiple sequence alignment of protein sequences of OsGRX20 with its related CPYC-type ones from other plant species was carried out. Amino acid sequence alignment shows that OsGRX20 carries domains (amino acids 144–225, 263–339) with high similarity to consensus sequences including the predicted CPFC active site of CPYC-type class at 153-156 amino acids at the N-terminus (Supplementary Figure S1), a typical dithiol isoform, that are able to catalyze protein disulfides and GSH-protein mixed disulfides (Lee et al., 2002; Morita et al., 2015). OsGRX20 shares a high identity with the putative GRXs from *O. brachyantha* (XP_006659693, 90%) and *Hordeum vulgare* (BAJ99765, 83%). Phylogenetic analysis revealed that OsGXR20 was not closely related to other members in CPYC-type class (Garg et al., 2010).

**FIGURE 1:**
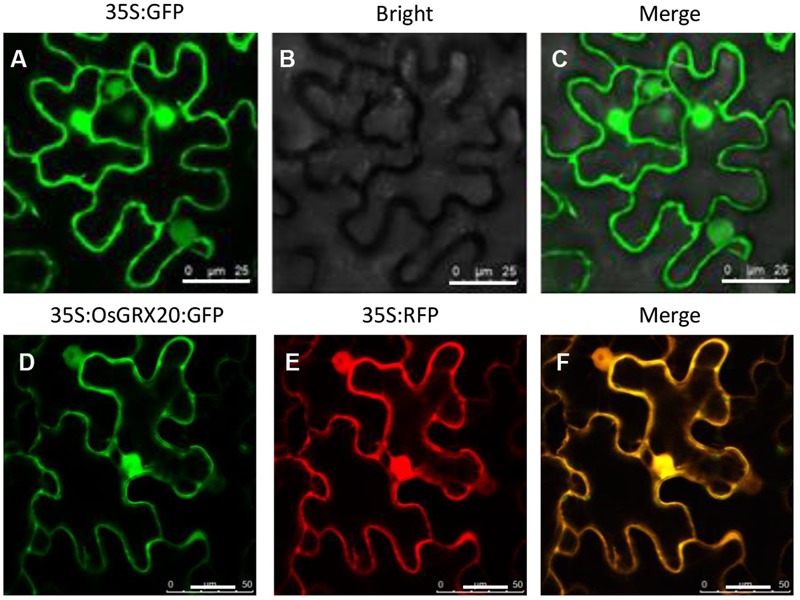
Analysis of the subcellular localization of OsGRX20 protein using a tobacco transient transformation system in tobacco epidermal cells. The vector 35S:GFP without any pieces of *OsGRX20* sequence as control under fluorescence channel **(A)**, bright-field channel **(B)**, and overlay **(C)**. 35S:OsGRX20:GFP construct **(D)** and 35S:RFP without *OsGRX20* sequence **(E)** under fluorescence channel. Merge of 35S:OsGRX20:GFP and 35S:RFP channels **(F)**. Agrobacterium carrying different vectors were infiltrated into *N. benthamiana* leaves. At 2 days post infiltration, the leaf epiderm was cut and examined by confocal laser scanning microscopy. The white bar represents 25 μm. This experiment was repeated three times with similar results.

To determine the subcellular localization of OsGRX20, N-terminal eGFP-fused *OsGRX20* was transiently expressed in epidermal cells of tobacco leaves through *Agrobacterium*-infiltration (Wu et al., 2012; Yang et al., 2016). The GFP-OsGRX20 signals are ubiquitously detected in the cytoplasm and nuclei (**Figure 1**).

### Expression Profile of *OsGRX20* Gene under Stress Conditions

**FIGURE 2:**
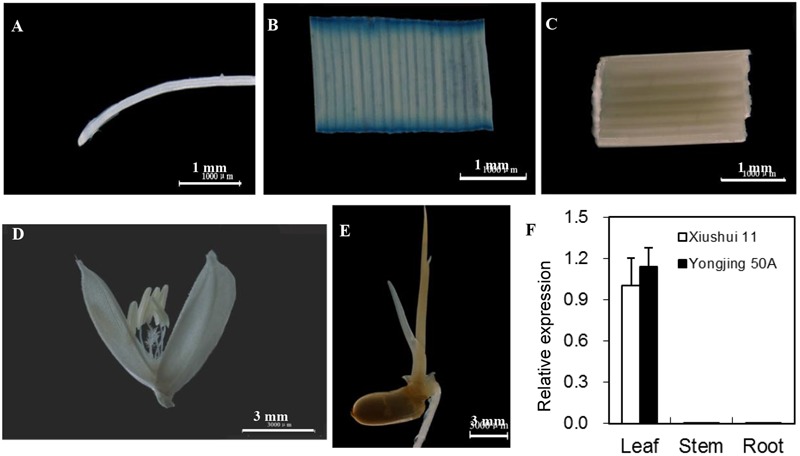
Spatial expression patterns of *OsGRX20*. Expression of GUS driven by the *OsGRX20* promoter of transgenic rice plant in root **(A)**, leaf **(B)**, stem **(C)**, flower **(D)** and germinated seed **(E)**. Scale bars are 1 mm **(A–C)** or 3 mm **(D,E)**. **(F)** Expression pattern revealed by qPCR. The expression level was firstly normalized using *β*-actin as an internal reference, and then made relative to the amount of corresponding mRNAs in leaf sample of Xiushui 11 variety. Bars represent means (three replicates) ± SD.

Garg et al. (2010) reported that *OsGRX20* mRNA was expressed at a high level in young and mature leaf based on microarray data. Here, qPCR analysis demonstrated that the transcript level of *OsGRX20* was high in leaf, but extremely low in root and stem (**Figure 2F**). The 2501-bp sequence upstream of the translational start codon ATG amplified from Xiushui 11 was identical with that from Yongjing 50A. To confirm the expression pattern, an expression vector of GUS protein driven by the *OsGRX20* promoter was constructed and transgenic plants were generated by transforming Yongjing 50A. The GUS histochemical staining demonstrated *OsGRX20* predominantly expressed in the leaf, very low in the root, stem, flower, or germinated seed (**Figures 2A–E**).

**FIGURE 3:**
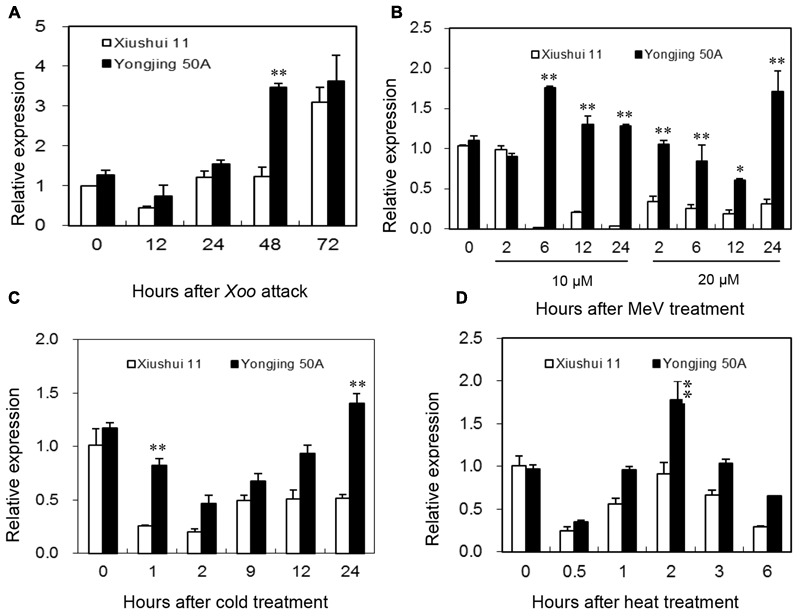
Expression of the *OsGRX20* gene in leaves in response to stress. Expression patterns of *OsGRX20* in two contrasting genotypes in response to *Xoo* strain-Zhe173 infection **(A)**, MeV treatments **(B)**, cold **(C)** or heat **(D)**. The transcript level was firstly normalized using *β*-actin as an internal reference, and then made relative to the amount of corresponding mRNAs in 0 h sample. Bars represent means (three replicates) ± SD. The asterisks indicate that a significant difference was detected between two genotypes (^∗^*p* < 0.05; ^∗∗^*p* < 0.01).

Expression patterns of *OsGRX20* in leaves between Xiushui 11 and Yongjing 50A in response to various oxidative stresses were compared by qPCR (**Figure 3**). Yongjing 50A is more resistant to *Xoo* strain-Zhe173 than Xiushui 11 (Huang et al., 2008; Zhang et al., 2015). As shown in **Figure 3A**, expression of *OsGRX20* gene was induced more rapidly in Yongjing 50A leaves inoculated with *Xoo* strain-Zhe173 than in Xiushui 11, 48 h for Yongjing 50A *vs* 72 h for Xiushui 11. And Yongjing 50A showed higher expression level of *OsGRX20* gene (*p* < 0.01) compared with Xiushui 11 at 48 h after Zhe173 attack (**Figure 3A**).

MeV that stimulates production of ROS in chloroplasts is often used to induce oxidative stress *in vivo* in plants (Fukao et al., 2011; Krieger-Liszkay et al., 2011). When the seedlings were treated with 10 μM MeV through roots, the transcript level of *OsGRX20* in Yongjing 50A showed a significant increase from 6 h to 24 h (*p* < 0.01), whereas that in Xiushui 11 was remarkably reduced (*p* < 0.01). At 20 μM MeV, the transcript level of *OsGRX20* was downregulated significantly in Xiushui 11 from 2 h to 24 h, whereas did not change until 12 h and upregulated at 24 h in Yongjing 50A (**Figure 3B**). It is noticeably that *OsGRX20* transcripts were significantly higher in Yongjing 50A than in Xiushui 11 at most time points of MeV treatments (**Figure 3B**). Similarly, Yongjing 50A showed higher expression level of *OsGRX20* gene (*p* < 0.01) compared with Xiushui 11 at at least one time point in the presence of cold (**Figure 3C**) and heat stress (**Figure 3D**). In addition, the transcript level of *OsGRX20* in Yongjing 50A was upregulated significantly (*p* < 0.01) in the early stage of 20% PEG-6000 (simulation of drought stress) and 100 mM NaCl treatment (Supplementary Figure S2A). When seedlings were sprayed with 20 μM 2,4-D, 2 mM SA, 100 μM JA and 100 μM ABA individually for 3 h, the transcript level of *OsGRX20* in Yongjing 50A leaves was significantly increased (*p* < 0.01) and relative expression levels were about 2, 3, 4, and 2-folds of control group, respectively (Supplementary Figure S2B). Together, the results indicate that *OsGRX20* might involve in the response to multiple stresses.

### Overexpression of *OsGRX20* in Rice Enhances Resistance to *Xoo* in the Susceptible Genotype

**FIGURE 4:**
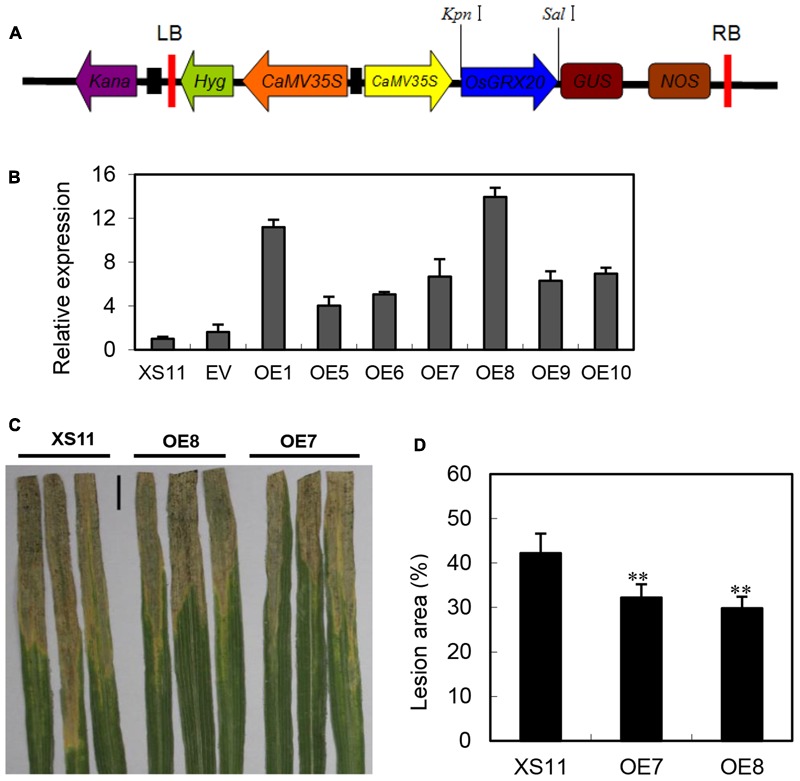
Overexpression of *OsGRX20* enhanced rice resistance to *Xoo* infection. **(A)** Schematic diagram of the *OsGRX20*-pCAMBIA1301 overexpression construct. **(B)** The transcript level of *OsGRX20* in T_1_ transgenic lines is relative to that in wild-type Xiushui 11 (XS11). Bars represent means (three to five replicates) ± SD. Lesion phenotype **(C)** and lesion area **(D)** in *OsGRX20-*overexpressing lines OE7 and OE8 compared with wild-type Xiushui 11 (XS11). The flag leaves at the booting stage were inoculated with *Xoo* strain-PXO66 for 21 days. The black bar represents 2 cm. The double asterisks indicate that a significant difference (*p* < 0.01) in the lesion area was detected between transgenic plants and the respective wild -type. Data represent mean ± SD from three independent biological replicates.

The expression of *OsGRX20* was significantly upregulated at both the transcript (**Figure 3A**) and protein level (Yu et al., 2008) by *Xoo* strain-Zhe173 infection, and also transcriptionally induced by signal molecules such as SA and JA (Supplementary Figure S2B). The aforementioned information prompted us to test the function of *OsGRX20* in rice defense responses. To investigate the role of *OsGRX20* in rice disease resistance to *Xoo, OsGRX20-*overexpressing transgenic lines driven by the CaMV35S promoter were generated in the background of Xiushui 11 (**Figure 4A**), which is susceptible to *Xoo* strains. All transgenic lines appeared to have normal development (Supplementary Table S2). qPCR analysis revealed that the transcript levels of *OsGRX20* in seven transgenic overexpressing lines (OE1, OE5, OE6, OE7, OE8, OE9, and OE10) were from 4- to 14-fold higher compared with wild-type or transgenic control (expressing only an empty vector) plants (**Figure 4B**). In these experiments, the most destructive *Xoo* strain-PXO99 was used to inoculate the flag leaves from T_3_ progeny of transgenic lines that *OsGRX20* is overexpressed at the booting stage. As shown in **Figures 4C,D**, the lesion areas of *OsGRX20*-overexpressing lines OE7 and OE8 are significantly reduced by 24 and 27% compared with wild-type Xiushui 11 (*p* < 0.01) at the 21st day post inoculation. The results exhibit that overexpression of *OsGRX20* enhance rice resistance to *Xoo* infection.

### Interference of *OsGRX20* Impairs Resistance to *Xoo* in the Resistant Genotype

**FIGURE 5:**
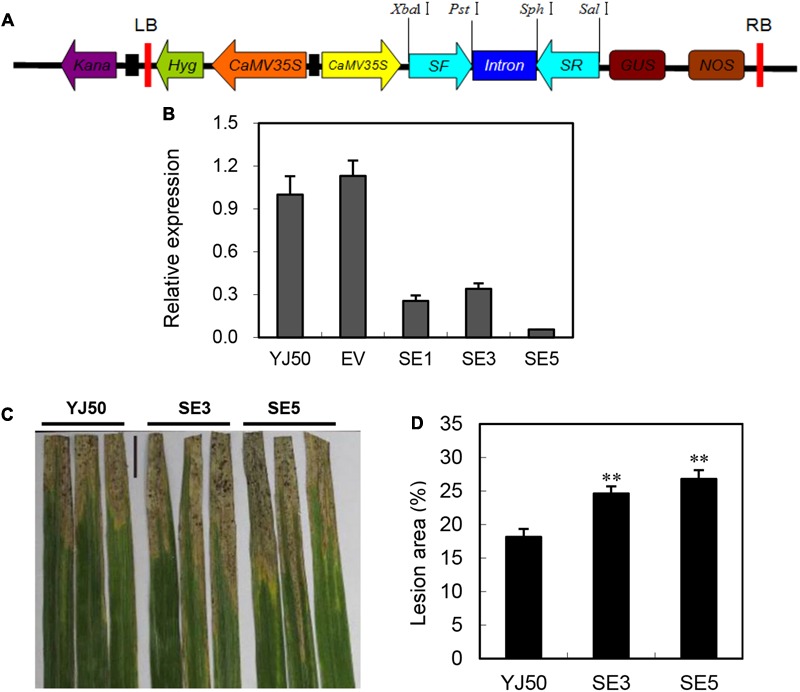
Interfering of *OsGRX20* increased susceptibility to *Xoo* infection. **(A)** Schematic diagram of the *OsGRX20*-RNAi construct. **(B)** The transcript levels of *OsGRX20* in T_2_ transgenic plants are relative to that in the wild-type Yongjing 50A (YJ50). Bars represent means (three to five replicates) ± SD. **(C)** Lesion phenotype. **(D)** Reduced resistance to *Xoo* in *OsGRX20-*interfering lines SE3 and SE5 compared with wild-type Yongjing 50A (YJ50). The flag leaves at the booting stage were inoculated with *Xoo* strain-PXO66 for 21 days. The black bar represents 2 cm. The double asterisks indicate that a significant difference (*p* < 0.01) in the lesion area was detected between transgenic plants and the respective wild-type. Data represent mean ± SD from three independent biological replicates.

To further confirm the role of *OsGRX20* in disease resistance, transgenic RNAi lines were generated in the background of Yongjing 50A (**Figure 5A**), which is resistance to *Xoo* strains. The sequence used for the RNAi harpin shares an extremely low identity with other members of *OsGRX* family. qPCR results show that the *OsGRX20* transcript levels are significantly reduced by 66–99% in three independent RNAi lines (SE1, SE3, and SE5) (**Figure 5B**). The lesion areas of the *OsGRX20*-interfering lines SE3 and SE5 are significantly increased to 35 and 29% compared with wild-type Yongjing 50A, respectively (**Figures 5C,D**). Suppressing *OsGRX20* increases the susceptibility of Yongjing 50A to *Xoo* strain-PXO99. The rice resistance to *Xoo* of *OsGRX20* transgenic plants is positively associated with the transcript level of *OsGRX20* (**Figures 4D,5D**). Together, the results demonstrate that *OsGRX20* participates in the positive regulation of bacterial disease resistance.

### *OsGRX20* Plays a Positive Role in Tolerance to MeV-Induced Oxidative Stress

**FIGURE 6:**
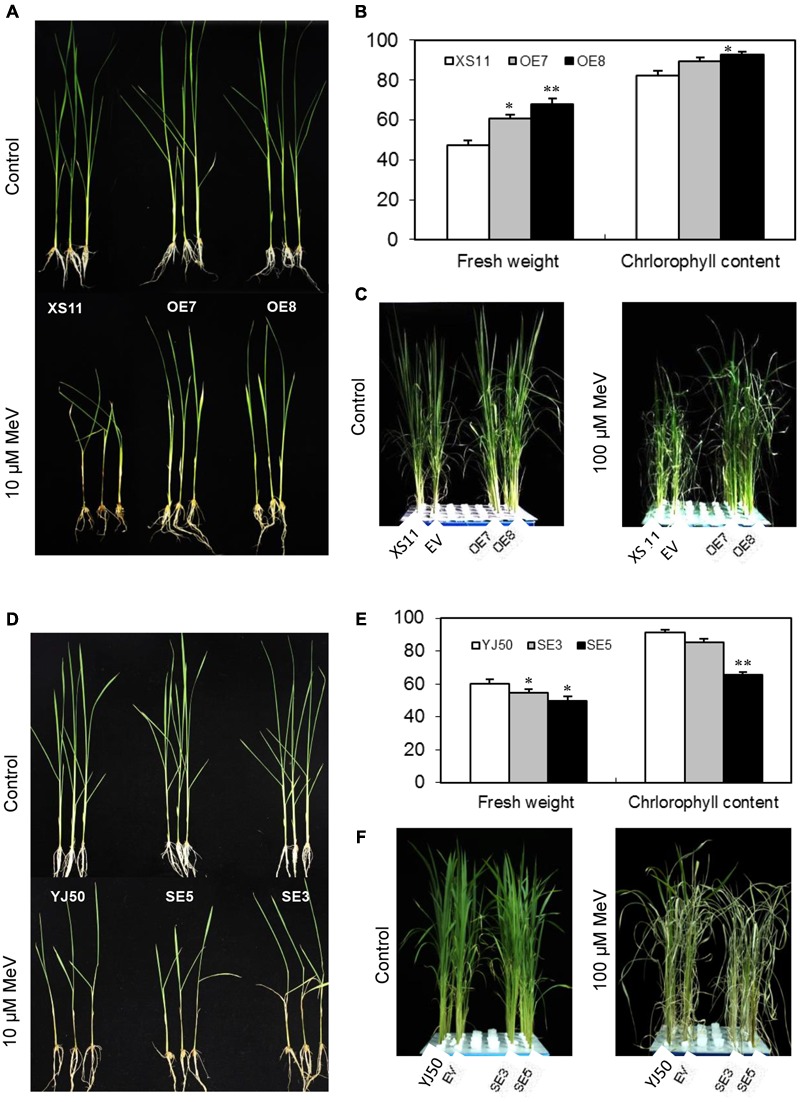
The role of *OsGRX20* in rice tolerance to MeV. **(A)** Growth of wild-type XS11 and *OsGRX20*-overexpression plants after 10 μM MeV treatment for 7 days. **(B)** Relative fresh weight and chlorophyll content of *OsGRX20*-overexpression and control plants. The seedlings at three-leaf stage were transferred to solution supplemented with 10 μM MeV for 7 days. All values are means (±SD) from two independent experiments (10 seedlings per experiment). The asterisks indicate that a significant difference (^∗^*p* < 0.05; ^∗∗^*p* < 0.01) was detected between transgenic plants and wild-type. **(C)** Changes of plant growth and leaf wilting in *OsGRX20*-overexpressed and control plants after sprayed with 100 μM MeV for 10 days. Seedlings grown in four rows in a plastic tank from left to right are wild-type Xiushui11 (XS11), expressing an empty vector (EV), overexpressing lines OE7 and OE8. **(D)** Phenotype of wild-type YJ50 and *OsGRX20*-RNAi plants after application of 10 μM MeV through roots for 7 days. **(E)** Relative fresh weight and chlorophyll content of *OsGRX20*-RNAi and control plants. **(F)** Changes of plant growth and leaf wilting in *OsGRX20*-RNAi and wild-type YJ50 plants are distinguishable after sprayed with 100 μM MeV for 10 days. Seedlings grown in four rows in a plastic tank from left to right are the wild-type Yongjing 50A (YJ50), expressing an empty vector (EV), RNAi lines SE3 and SE5.

Application of MeV through root suppressed seedling growth of both genotypes (Supplementary Figure S3A), but the reduction in dry weight was more significant (*p* < 0.05) in Xiushui 11 at the concentration of 20 μM for 7 days (Supplementary Figure S3B). The roots and stem bases of Xiushui 11 seedlings grown in MeV solution became darker than Yongjing 50A (Supplementary Figure S3A). Thus, rice Yongjing 50A is insensitive to MeV treatment than Xiushui 11. T_2_ progeny from lines OE7 and OE8, and wild-type plants were incubated with 10 μM MeV for 7 days. As shown in **Figure 6A**, *OsGRX20*-overexpressing plants display robust growth, whereas the wild-type plants exhibit retarded growth and black stems after the treatment. The relative fresh weight of the *OsGRX20*-overexpressing plants is significantly higher (*p* < 0.05 or *p* < 0.01) compared with the wild-type (**Figure 6B**). A significant difference (*p* < 0.05) in the relative chlorophyll content between *OsGRX20*-overexpressing line OE8 and wild-type seedlings is also observed (**Figure 6B**). In addition, the leaves of wild-type Xiushui 11 and transgenic control plants are severely wilt and damaged; in contrast, the leaves of *OsGRX20*-overexpressing plants are still green and healthy when the leaves were sprayed with 100 μM MeV for 10 days (**Figure 6C**). These results exhibit that overexpression of *OsGRX20* enhance tolerance to MeV-induced oxidative stress.

After incubating the T_2_ progeny of RNAi lines SE3 and SE5 with 10 μM MeV for 7 days, *OsGRX20*-interfering plants show reduced growth compared with wild-type Yongjing 50A (**Figure 6D**). As shown in **Figure 6E**, the relative fresh weights of two *OsGRX20*-interfering lines are significantly lower (*p* < 0.05) than the wild-type seedlings. The relative chlorophyll content of SE5 leaves which displayed the lowest *OsGRX20* expression is decreased significantly (*p* < 0.01) compared with the wild-type (**Figure 6E**). Accordingly, the RNAi seedlings demonstrate growth retardation; more severe leaf wilting as compared to wild-type and transgenic control seedlings when the leaves were sprayed with 100 μM MeV for 10 days (**Figure 6F**), indicating that suppressing of *OsGRX20* increase the susceptibility of Yongjing 50A to MeV treatment.

### *OsGRX20* Mediated the Tolerance against Salt-Induced Oxidative Stress

**FIGURE 7:**
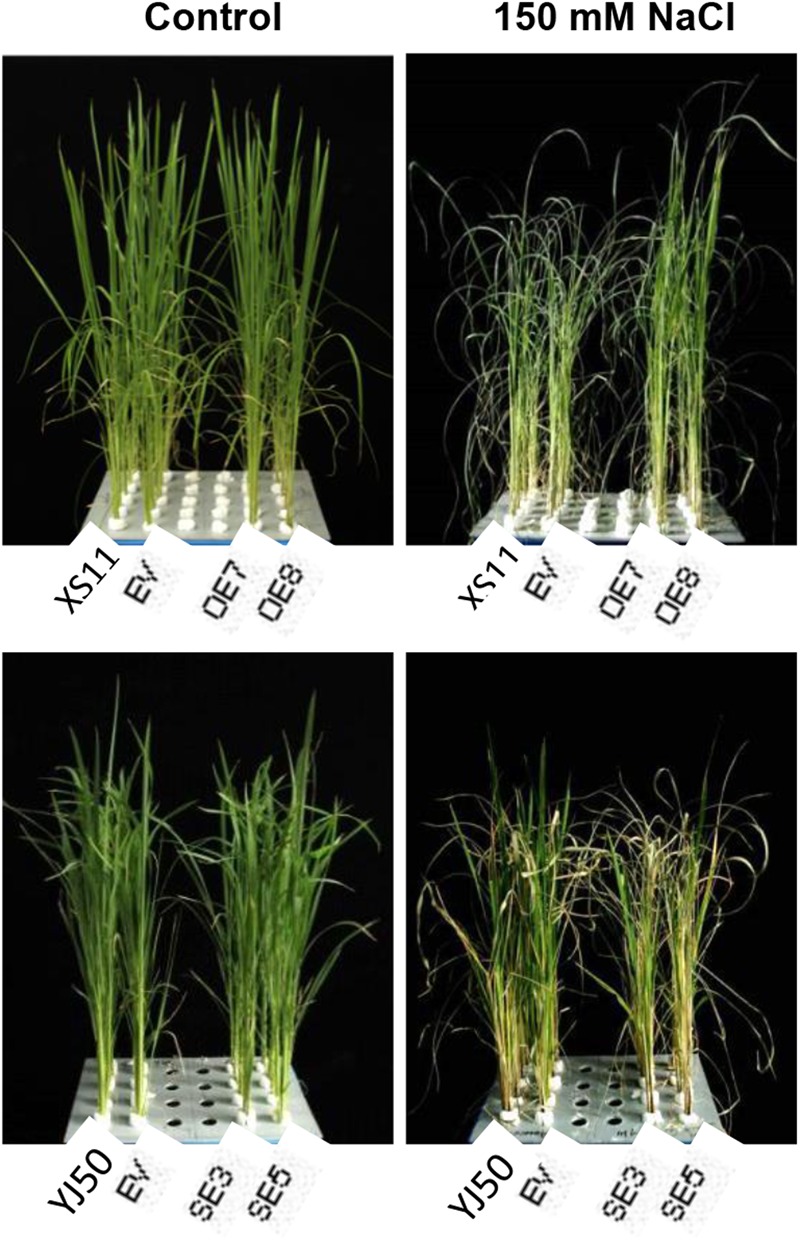
Effects of *OsGRX20* overexpression and RNAi on the growth and wilting degree under salt stress. The seedlings at five-leaf stage were transferred to 150 mM NaCl for 0 day **(left)** and for 8 days **(right)**. Seedlings grown in four rows in a plastic tank from left to right are the wild-type Xiushui11 (XS11), expressing an empty vector (EV), overexpressing lines OE7 and OE8 **(top)**; wild-type Yongjing 50A (YJ50), expressing an empty vector (EV), RNAi lines SE3 and SE5 **(bottom)**.

Given the facts that *OsGRX20* is a stress-responsive gene and induced by salinity (Supplementary Figure S2A), it might be essential for rice seedlings’ tolerance to salt stress. To evaluate the role of *OsGRX20* in the rice tolerance to salt treatment, the changes in seedling growth of overexpressing and suppressing the gene in a solution of 150 mM NaCl for 8 days were studied. As shown in **Figure 7**, the seedlings of *OsGRX20-*overexpressing lines OE7 and OE8 wilt lightly and grow better, and exhibit enhanced tolerance to salt stress relative to wild-type Xiushui 11 or transgenic control phenotypically (top panel). In contrast, the RNAi lines SE3 and SE5 with severely wilted and yellow leaves display significantly increased sensitivity compared with wild-type Yongjing 50A or transgenic control (bottom panel).

### ROS Production and Detoxification under MeV Stress

**FIGURE 8:**
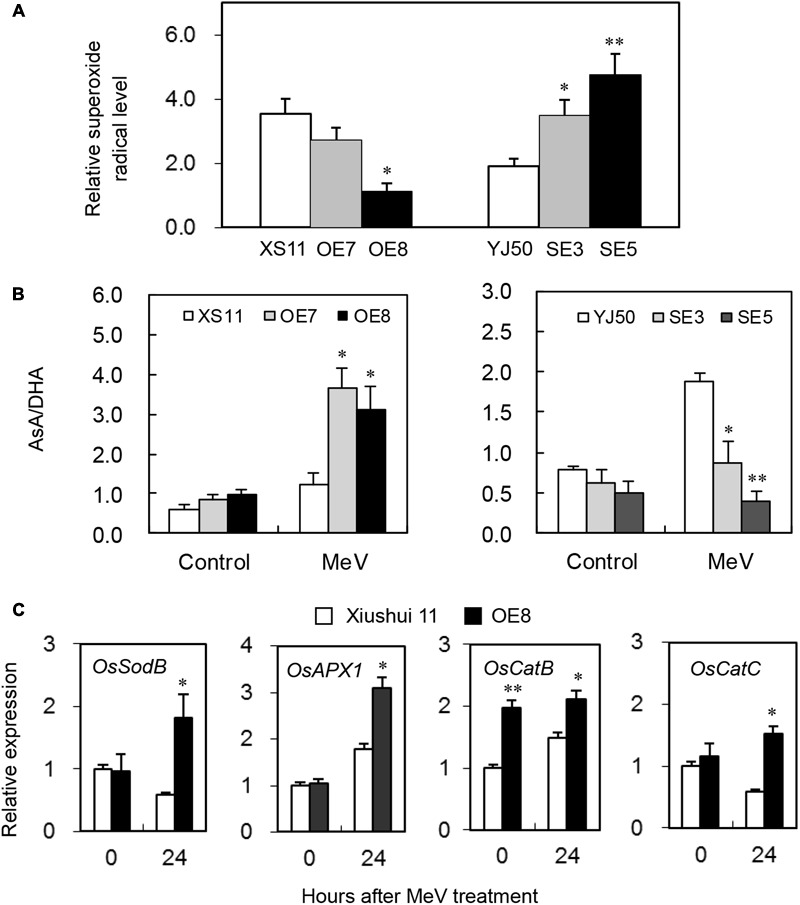
Modulating *OsGRX20* expression influenced levels of superoxide radicals, AsA/DHA and gene transcripts associated with ROS detoxification after MeV treatment. Five-leaf plants from wild-type Xiushui11 (XS11) and Yongjing 50A (YJ50), *OsGRX20*-overexpression lines OE7 and OE8, and RNAi lines SE3 and SE5 were incubated in 10 μM MeV for 24 h, and the aerial tissue was subjected to analysis. **(A)** Relative superoxide radical level was calculated by comparison to the non-treated seedlings of individual genotypes. **(B)** The AsA/DHA ratio under control condition and after 24-h treatment. **(C)** Relative mRNA levels of four genes associated with ROS detoxification in *OsGRX20*-overexpression line OE7 and wild-type Xiushui 11. Data represent mean ± SD from three independent biological replicates, and an asterisk indicates a significant difference between wild-type vs. transgenic plants (^∗^*p* < 0.05; ^∗∗^*p* < 0.01).

To evaluate whether *OsGRX20* plays a role in the capacity of plants to detoxify ROS, the level of superoxide radicals was assessed. As shown in **Figure 8A**, the two RNAi lines SE3 and SE5 show higher relative level of superoxide radicals compared with wild-type Yongjing 50A. In contrast, the *OsGRX20-*overexpresing line OE8 has a significantly lower relative level of superoxide radicals compared with wild-type Xiushui 11. It was then investigated whether changes in ROS levels correlated with alterations in the levels and redox state of ASC. The ASC/DHA ratio did not differ between the wild-type and transgenic plants before MeV treatment. Interestingly, the ASC/DHA ratio in the two *OsGRX20-*overexpresing lines was significantly higher than in Xiushui 11 plants after 24-h stress, whereas that in the two RNAi lines was significantly lower than in Yongjing 50A (**Figure 8B**).

The plants are equipped with constitutive and inducible isoforms of SOD to detoxify superoxide, and enzymes from the ASC- and GSH-dependent systems to detoxify H_2_O_2_ (Laporte et al., 2012). Ascorbate peroxidase (APX), SOD, and catalase (CAT) are major enzymes that detoxify superoxide and H_2_O_2_ under stressed conditions in rice plants (Fukao et al., 2011). Four genes coding key components of the cytosolic antioxidant/ROS scavenging system, *OsSodB* (LOC_Os06g05110), *OsAPX1* (LOC_Os03g17690), *OsCatB* (LOC_Os06g51150), and *OsCatC* (LOC_Os03g03910), were expressed significantly higher in *OsGRX20-*overexpresing plants than in Xiushui 11 after 24-h treatment of MeV (**Figure 8C**), whereas *OsSodA* (LOC_Os05g25850), *OsAPX2* (LOC_Os07g49400), and *OsCatA* (LOC_Os02g02400) did not show different expression between OE7 and Xiushui 11 plants both before and after the stress (data not shown). The results here support a role for OsGRX20 as an important component of the cellular ROS-scavenging/antioxidant system, thereby improving tolerance to multiple stresses.

## Discussion

Multiple stresses including salinity, drought, heat, cold, and pathogen attack augment cellular ROS production. Elevated ROS serve as redox signaling molecules to initiate anti-oxidative adaptation mechanism to the stresses (Fukao et al., 2011). MeV stimulates superoxide production within the thylakoids of tobacco leaves by radical-induced auto-oxidation, which have been generated by electrons from PSI (Krieger-Liszkay et al., 2011). However, the excessive accumulation of ROS can oxidize DNA, proteins and lipids, thereby resulting in cellular damage and genomic instability. During the detoxification of ROS, scavengers must be regenerated by scavenger recovery proteins, i.e., GRXs or TRXs. There is mounting evidence showing that GRXs possess anti-oxidative activities in plants directly and indirectly (Cheng et al., 2006; Laporte et al., 2012). In present study, we characterized a rice typical CPYC-type *GRX, OsGRX20*, and confirmed that *OsGRX20* gene contributes to seedling tolerance to oxidative challenge induced by biotic and abiotic stresses by using the transgenic plants overexpressing and interfering *OsGRX20* gene, respectively.

Although *GRX* genes of CPYC-type class are conserved among prokaryotes and eukaryotes, OsGRX20 is not closely related to six other members in the phylogenetic tree, and no ortholog was identified in *Arabidopsis* (Garg et al., 2010). Each of seven *OsGRX* genes (*OsGRX9, OsGRX10, OsGRX12, OsGRX14, OsGRX18, OsGRX20*, and *OsGRX21*) was differentially expressed in various tissues or developmental stages and their transcript level was differentially regulated by environmental stimuli (Garg et al., 2010), suggesting that these seven genes are functionally complementary instead of redundant. Only limited microarray datasets for *OsGRX20* expression were available (Garg et al., 2010). Here, both qPCR and histochemical GUS analyses demonstrated that *OsGRX20* expression was predominant in the leaves (**Figure 2**).

Gene expression is largely controlled by its promoter (Mou et al., 2013). A 2501-bp promoter sequence of *OsGRX20* gene from Xiushui 11 was identical with that from Yongjing 50A. In this sequence, several putative *cis*-elements associated with stress responses including heat shock element, low temperature response element (Mani et al., 2015), MYB binding site (Smita et al., 2015), dehydration-responsive element (Maruyama et al., 2012) and W-box (Smita et al., 2015), were identified by using the PlantCARE and PLACE databases. Consistently, the transcript levels of *OsGRX20* significantly responded to *Xoo* attack, MeV, heat, cold, salt, and PEG-6000 treatments in our expression studies (**Figure 3** and Supplementary Figure S2A). The hormone-responsive elements were also identified in the promoter region of *OsGRX20* gene: ABREs or ABA-responsive elements (Maruyama et al., 2012), TGACG- or CGTCA-motif responsible for JA-responsiveness (Mani et al., 2015), AuxRE for auxin response (Smita et al., 2015), TCA-element for the response to SA and pathogen infection (Mou et al., 2013). Furthermore we found that *OsGRX20* transcript showed a significant increase in response to exogenous application of SA, JA, ABA, and 2,4-D (Supplementary Figure S2B). *OsGRX20* gene responded to multiple stresses and exogenous hormones, suggesting that *OsGRX20* is involved in rice tolerance to multiple stresses. To our knowledge, this study was the first to confirm the function of *GRX* family in plant defense against to biotic stress. Given the identical sequences of *OsGRX20* genomic, cDNA and promoter in two contrasting genotypes, we speculate that a regulatory gene or small RNA might control the expression of the gene and subsequently result in the different effect of *OsGRX20* expression in tolerant and sensitive genotypes under various stresses.

Multiple members of GRXs are known to be sublocalized in the cytosol, nuclei, nucleocytoplasm, chloroplasts, mitochondria and the secretory pathway in higher plants (Rouhier, 2010; Hong et al., 2012; Laporte et al., 2012; Sharma et al., 2013). It has been experimentally demonstrated that OsGRX14 was localized in subcellular organelles of the secretory pathway including the Golgi apparatus, endoplasmic reticulum, and plasma membrane (Morita et al., 2015). The present results manifested that the OsGRX20-GFP fusion protein was localized in the nucleus and cytoplasm of tobacco cells (**Figure 1**). Previously, the *Arabidopsis* ROXY1 was found to be localized in nucleus, which was crucial for interaction with TGA family transcription factors in regulating petal development (Li et al., 2009). We speculated that localization of OsGRX20 in the nucleus and cytosol may also participate in regulating the expression of stress-responsive gene through interaction with transcription factors. One may argue why a nucleo-cytoplasmic OsGRX provides such a high level of protection against oxidative stress in the chloroplasts induced by MeV. First of all, the organelles of plant cells are not isolated compartments and they are closely communicated by exchanging numerous signals and biomolecules. Two of numerous examples to support this statement are (1) activation of the tobacco SIPK/Ntf4/WIPK signaling cascade in cytoplasm and/or nucleus by pathogen actively promotes the generation of ROS in chloroplasts, which plays a key role in induction of hypersensitive response cell death in plants (Liu et al., 2007); (2) ROS generated in different compartments and cell types can triggers SA biosynthesis in chloroplasts (Herrera-Vásquez et al., 2015); Secondly, ROS and reactive nitrogen species have been shown to serve as diffusible intra- and intercellular signals for activation of various physiological reactions in plants (Neill et al., 2002). The diffusible nature of ROS implies that the ROS generated in chloroplast most likely will influence the redox state in cytoplasm or nucleus to certain extent. Therefore, the coordinated ROS detoxifications from both chloroplastic and nucleo-cytoplasmic GRX/TRX antioxidant systems should additively alleviate the toxic effect of chloroplast-generated ROS to the cells.

GRXs can reduce H_2_O_2_ and DHA directly (Sha et al., 1997; Lee et al., 2002; Morita et al., 2015). OsGrxC2;2 contains two conserved cysteine residues that are essential for its enzymatic activities (Lee et al., 2002). In the presence of GSH as an electron donor, OsGrxC2;2 reduces the disulfide bonds of other proteins. The oxidized OsGrxC2;2 protein is reduced subsequently via the GSH system. OsGrxC2;2 itself can function as a thioltransferase and a DHAR (Sha et al., 1997) as well as a GSH-dependent peroxidase (Lee et al., 2002). The increase in OsGrxC2;2 activities results in the elevation of ROS-scavenging capacity or redox regulation of target proteins (Morita et al., 2015). OsGRX20 protein also has two conserved cysteine residues at position 153 and 156 (Supplementary Figure S1). The increased tolerance or resistance to multiple oxidative stress in transgenic plants by the overexpression of *OsGRX20* is probably achieved via the elevation of ROS-scavenging capacity, reducing target proteins, and protecting thiol groups on other enzymes as OsGrxC2;2 does (Lee et al., 2002; Morita et al., 2015). Similar to what have observed for the *Arabidopsis ROXY18/GRXS13.2* overexpressing plants (Laporte et al., 2012), overexpressing *OsGRX20* in rice increased ASC/DHA ratio (**Figure 8B**), indicating both CC-type and CPYC-type GRXs could regulate cellular redox state through modulating DHAR activity either directly or indirectly. Interestingly, it has been shown that DHAR can be *S*-glutathionylated (Dixon et al., 2005) and thus raises the possibility that GRXs regulate DHA level through *S*-deglutathionylation.

Investigation of catalytic activity, target substrates and interactors of OsGRX20 can shed light on the mechanism for how this protein functions in stress tolerance. Transgenic plants at T_3_ generation showed agronomic traits statistically similar to wild-type including plant height, tiller number, panicle length and kernel weight per plant except for hundred-grain weight (Supplementary Table S2). These results provide useful information to us for further studying the molecular mechanism of rice in response to environmental stresses and for genetic engineering of crops to improve their disease resistance and stress tolerance.

## Conclusion

*OsGRX20* encodes a protein for CPYC-type GRX with CPFC active site. OsGRX20 positively regulates plant responses to bacteria attack and multiple abiotic stresses.

## Author Contributions

LY and CW designed the study. XN carried out vector construction, rice transformation and phenotype assays experiments. YS participated in the subcellular localization and histochemical experiments. WZ performed the bioinformatic analyses. HH participated in the growing of transgenic rice. MS assisted in conducting the experiments, especially prokaryotic expression. LY conceived the project and wrote the manuscript. All authors read and approved the manuscript.

## Conflict of Interest Statement

The authors declare that the research was conducted in the absence of any commercial or financial relationships that could be construed as a potential conflict of interest.
