# Supplementary material for: A Rice CPYC-Type Glutaredoxin OsGRX20 in Protection against Bacterial Blight, Methyl Viologen and Salt Stresses
Source: Front Plant Sci. 2018 Feb 9;9:111. doi: 10.3389/fpls.2018.00111 (PMC5811478; doi:10.3389/fpls.2018.00111)
Supplement: Supplementary file 1 [file Table_1.PDF]

Supplementary Table S1. Sequences of primers used for amplification, expression analysis and vector construction.

Primer Name	Sequence (5'---3')	Usage
GRX20-F	GATTCCTCCTGTGCTGTGCG	Full-length amplification
GRX20-R	AAAACAAGTAAACAGGGAGT	
GRX20q-F	ACCTCCACCTCTGATAAGCC	qPCR
GRX20q-R	CACTGCCCTATCTTTGATGACTC	
Actin-F	TTGCCAAGGCTGAGTACGACGA	Reference gene
Actin-R	AAACAAGCAGGAGGACGGCGAT	
GRX20p-F	GGAATTCCCAAAGGACAAAGAGACAT (*Eco*RI)	GUS reporter vector
GRX20p-R	ACGCGTCGACCGAAGACGACGCAACAA (*Sal*I)	
GRX20OE-F	CGGGGTACCATGGCGGCCACCGCGCTC (*Kpn*I)	Overexpression vector
GRX20OE-R	ACGCACGCGTCGACTGAGAAGTAGAAGGGATTC (*Sal*I)	
GRX20SE-1F	GCTCTAGAACAACTATAACAGCAGGGC-3′ (*Xba*I)	RNA interference vector
GRX20SE-1R	AACTGCAGTGAGAAGTAGAAGGGATTC-3′ (*Pst*I)	
GRX20SE-2F	ACGCGTCGACACAACTATAACAGCAGGGC-3′ (*Sal*I)	
GRX20SE-2R	ACATGCATGCTGAGAAGTAGAAGGGATTC-3′ (*Sph*I)	
GRX20s-R	CGGGGTACCAGCCGCATATGTGGCCCTC (*Kpn*I)	
OsAPX1-F	AGGTGCCACAAGGAAAGATCTGGT	qPCR
OsAPX1-R	TCAGCAGGGCTTTGTCACTAGGA	
OsAPX2-F	TGGGAAGATGCCACAAGGAGAGAT	qPCR
OsAPX2-R	TCCGCAGCATATTTCTCCACCAGT	

OsSodA1-F	ATCTGGATGGGTGTGGCTAGCTTT	qPCR
OsSodA1-R	AGTACGCATGCTCCCAGACATCAA	
OsSodB-F	TCCGCCGTATAAACTTGATGCCCT	qPCR
OsSodB-R	TGGGTTGCCGTTGTTGTATGCTTC	
OsCatA-F	CAACCGCAACGTCGACAACTTCTT	qPCR
OsCatA-R	TTCACCGGCAGCATCAGGTAGTTT	
OsCatB-F	GCTTGCTTTCTGCCCAGCGATAAT	qPCR
OsCatB-R	AAATAGTTTGGGCCAAGACGGTGC	
OsCatC-F	AGAAGGTGGTGATTGCCAAGGAGA	qPCR
OsCatC-R	TCTCTTGATGAACCGGTCTTGCCT	
