# Supplementary material for: A Rice CPYC-Type Glutaredoxin OsGRX20 in Protection against Bacterial Blight, Methyl Viologen and Salt Stresses
Source: Front Plant Sci. 2018 Feb 9;9:111. doi: 10.3389/fpls.2018.00111 (PMC5811478; doi:10.3389/fpls.2018.00111)
Supplement: Supplementary file 2 [file Table_2.PDF]

Supplementary Table S2. Agronomic traits of transgenic plants in the fields.

Agronomic trait	Xiushui11	EV	OE7	OE8	Yongjing 50A	EV	SE3	SE5
Plant height (cm)	78.6±1.5	80.0±1.7	79.8±1.1	80.0±1.5	70.9±1.5	71.4±2.1	70.5±1.2	69.8±0.6
Tiller number	11±1	12±2	12±1	12±1	11±2	12±1	12±2	12±2
Panicle length (cm)	20.1±1.4	20.9±0.7	21.1±2.5	20.6±1.9	16.8±0.5	16.8±0.5	16.4±0.5	16.4±0.5
Kernel weight per plant (g)	18.79±2.05	17.62±0.86	18.82±0.86	18.25±0.56	15.75±1.46	14.82±1.62	14.18±1.50	15.71±0.95
Hundred-grain weight (g)	2.78±0.03	2.77±0.10	3.00±0.13*	3.07±0.09*	2.45±0.06	2.39±0.07	2.19±0.10*	2.18±0.08*
