# Supplementary material for: A Rice CPYC-Type Glutaredoxin OsGRX20 in Protection against Bacterial Blight, Methyl Viologen and Salt Stresses
Source: Front Plant Sci. 2018 Feb 9;9:111. doi: 10.3389/fpls.2018.00111 (PMC5811478; doi:10.3389/fpls.2018.00111)
Supplement: Supplementary file 3 [file Image_1.PDF]

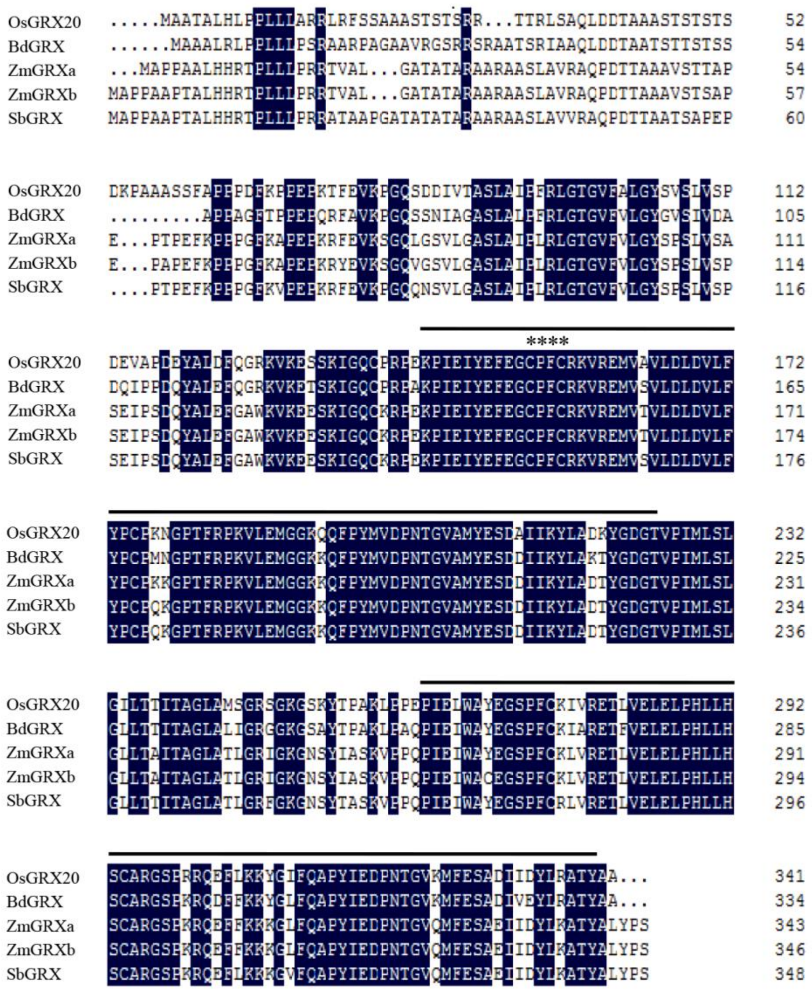

Supplementary Figure S1. Amino acid sequence alignment between OsGRX20 and other GRXs sequences with Clustal-W software. BdGRX is from *Brachypodium distachyon* (GenBank Accession Number KQJ99361), ZmGRXa and ZmGRXb from *Zea mays* (XP_008664566 and XP_008664567), and SbGRX from *Sorghum bicolor* (XP_002445682). Identical residues are shaded *dark blue*. Two conserved motifs are overlined, and four active site residues are indicated with *asterisks.*
