# Supplementary material for: A Rice CPYC-Type Glutaredoxin OsGRX20 in Protection against Bacterial Blight, Methyl Viologen and Salt Stresses
Source: Front Plant Sci. 2018 Feb 9;9:111. doi: 10.3389/fpls.2018.00111 (PMC5811478; doi:10.3389/fpls.2018.00111)
Supplement: Supplementary file 4 [file Image_2.PDF]

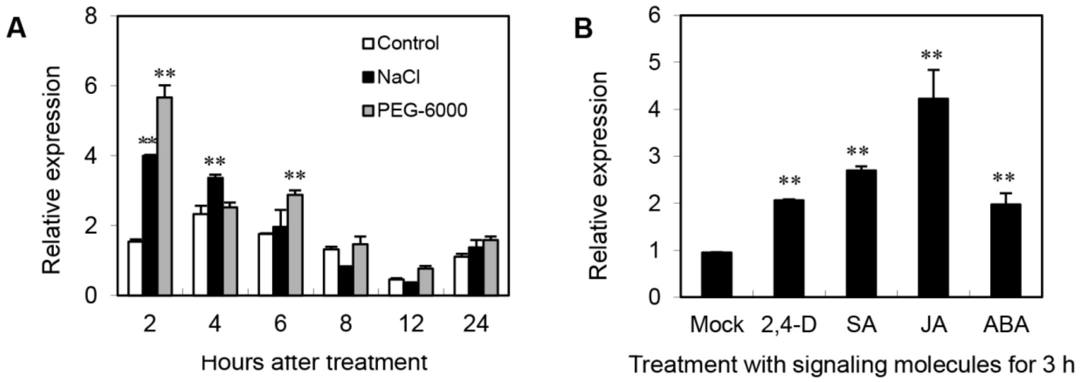

Supplementary Figure S2. The relative expression of *OsGRX20* in tolerant Yongjing 50A after incubated with 20% PEG-6000 and 150 mM NaCl (**A**), or sprayed with 20 μM 2,4-D, 2 mM SA, 100 μM JA and 100 μM ABA for 3 h (**B**). The expression level was firstly normalized using *β*-actin as an internal reference, and then made relative to the amount of corresponding mRNAs in 0 h sample. Bars represent means (three replicates) ± SD. The double asterisks indicate that a significant difference was detected between treated group and control group (p<0.01).
