# Supplementary material for: A Rice CPYC-Type Glutaredoxin OsGRX20 in Protection against Bacterial Blight, Methyl Viologen and Salt Stresses
Source: Front Plant Sci. 2018 Feb 9;9:111. doi: 10.3389/fpls.2018.00111 (PMC5811478; doi:10.3389/fpls.2018.00111)
Supplement: Supplementary file 5 [file Image_3.PDF]

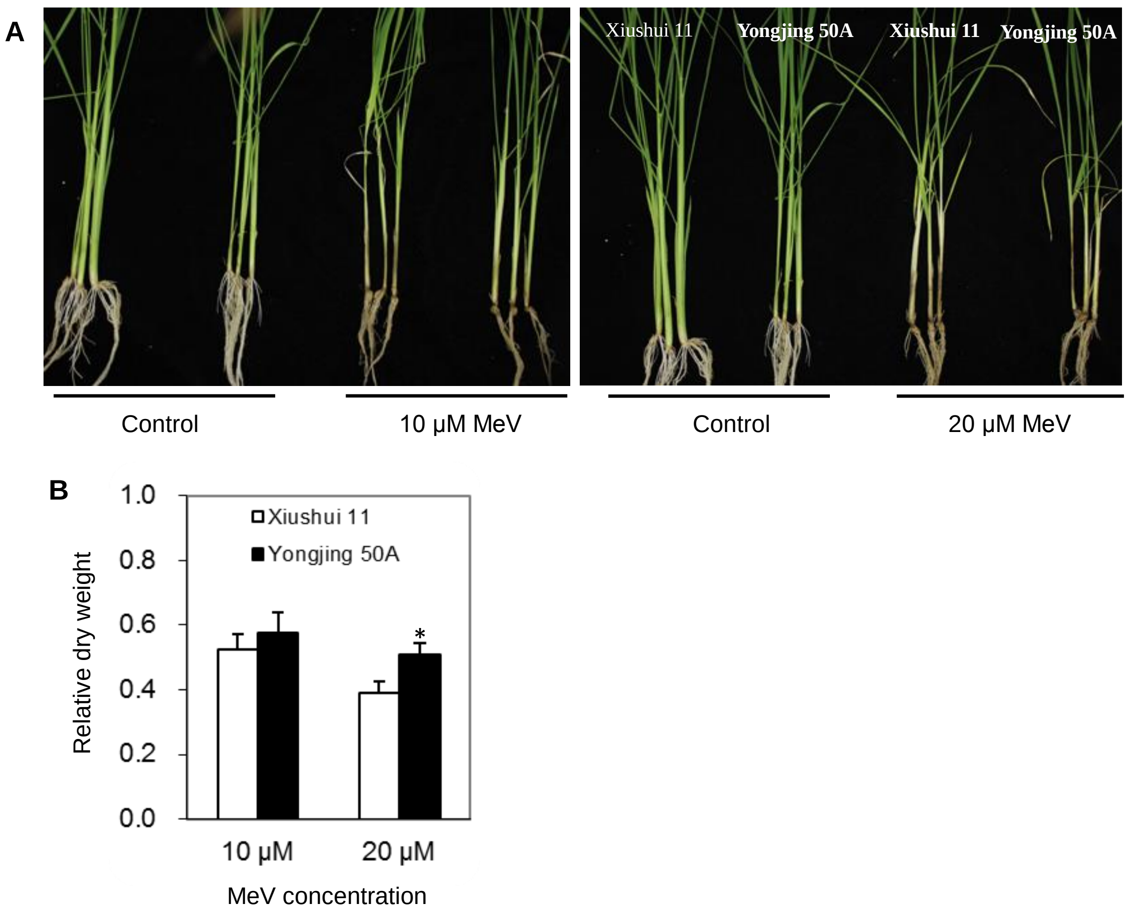

Supplementary Figure S3. Differential responsiveness between rice genotypes Xiushui 11 and Yongjing 50A to MeV treatments. Phenotypes **(A)** and the relative dry weight in the treated versus control plants **(B)** at the 7th day after transferred to a 10 or 20 μM MeV solution. The experiment was repeated three times with similar results. Bars represent means (three replicates) ± SD. The asterisks indicate that a significant difference (p<0.05) was detected between two genotypes.
